# Gold Nanoparticle Clusters in Quasinematic Layers of Liquid-Crystalline Dispersion Particles of Double-Stranded Nucleic Acids 

**Published:** 2012

**Authors:** Yu.M. Yevdokimov, V.I. Salyanov, E.I. Katz, S.G. Skuridin

**Affiliations:** Engelhardt Institute of Molecular Biology, Russian Academy of Sciences, Vavilova Str., 32, Moscow, Russia, 119991; Landau Institute for Theoretical Physics, Russian Academy of Sciences, Kosygina Str. 2, Moscow, Russia, 119334

**Keywords:** DNA, poly(I)×poly(C), liquid-crystalline dispersions of nucleic acids, gold nanoparticles, circular dichroism, absorption spectroscopy, abnormal optical activity, surface plasmon resonance, structure of biopolymer lyotropic liquid crystals, cytotoxicity of nanoparticles

## Abstract

The interaction between gold nanoparticles and particles of cholesteric liquid-crystalline dispersions formed by double-stranded DNA and poly(I)×poly(C) molecules is considered. It is shown that small-sized (~ 2 nm) gold nanoparticles induce two different structural processes. First, they facilitate the reorganization of the spatial cholesteric structure of the particles into a nematic one. This process is accompanied by a fast decrease in the amplitude of an abnormal band in the CD spectrum. Second, they induce cluster formation in a “free space” between neighboring nucleic acid molecules fixed in the structure of the quasinematic layers of liquid-crystalline particles. This process is accompanied by slow development of the surface plasmon resonance band in the visible region of the absorption spectrum. Various factors influencing these processes are outlined. Some assumptions concerning the possible mechanism(s) of fixation of gold nanoparticles between the neighboring double-stranded nucleic acid molecules in quasinematic layers are formulated.

## INTRODUCTION 

Metal and metal oxide nanoparticles are known to be characterized by their inherent ability to exhibit specific properties depending on the nanoparticle’s size. These properties of nanoparticles differ substantially from those typical of a “bulky” sample of the initial material. Nano-sized gold (Au) nanoparticles that are used both for research and applied purposes [1] (in particular, for diagnosis and treatment of certain diseases [2, 3]) are among the most vivid examples of the existence of such differences. Although the *in vitro* and *in vivo* cytotoxicity of Au nanoparticles has been investigated by several research teams, the data pertaining to the biological effects induced by Au nanoparticles are rather controversial [4, 5]. It is quite possible that the reason for this is that different biological systems have been used to study the effect of nanoparticles; in this case, it is difficult to compare their action mechanisms. 

The data [3, 6] provide a background to assume that the *in vitro* and * in vivo* action of Au nanoparticles on spatially arranged DNA structures is similar to that of molecules that possess mutagenic activity. Particles of DNA cholesteric liquid-crystalline dispersion (CLCD) are known to be among the structures that model certain spatial features of DNA within biological objects [7]. Indeed, the physicochemical features of DNA CLCD particles indicate some properties, which are characteristic of Protozoan chromosomes (e.g., chromosomes of Dinoflagellate, etc.) and DNA-containing bacteriophages [8–10]. 

Hence, DNA CLCD is a system of undoubted interest both in terms of nano- and biotechnologies. 

When studying the effect of Au nanoparticles on various biological macromolecules and systems, several facts should be borne in mind. Au nanoparticles, especially the small-sized ones, tend to spontaneously aggregate in water–salt solutions [1, 11, 12] and to form various complexes and aggregates with the solution components and dissolved macromolecules [13–16]. This process, accompanied by the approaching of neighboring Au nanoparticles, results not only in the enhancement of the so-called surface plasmon resonance (SPR) band typical of individual Au nanoparticles, but also in excitation of the collective vibrations of the electronic system and interaction between the neighboring “plasmons.” The latter effect, known as plasmon overlapping, is accompanied [1, 17, 18] by a shift of the SPR band toward the shorter or longer wavelengths of the absorption spectrum depending on a number of parameters (interparticle distance, size and shape of the resulting aggregates, dielectric permittivity of the medium [19, 20], existence of “interlayers” between the neighboring Au nanoparticles [21, 22], etc.). It is obvious that the complex formation (and possible aggregation of neighboring Au nanoparticles) is dependent on the concentration and charge of Au nanoparticles, their size, and the properties of the solvent components. This means that when studying the interaction between Au nanoparticles and biopolymer molecules, control experiments are to be carried out which would prove the absence of “parasitic” optical effects induced by the formation of nonspecific aggregates between Au nanoparticles and the solvent components under the conditions used. 

Hence, this work was aimed not only at proving the fact that there are no unnspecific aggregates between Au nanoparticles and the solvent components, but also at analyzing the interaction between Au nanoparticles and the double-stranded DNA molecules fixed in the spatial structure of the CLCD particles formed by phase exclusion of DNA molecules from water–salt solutions. 

## MATERIALS AND METHODS 

Colloid gold solutions (hydrosols) containing spherical nanoparticles of different sizes were used in this study. Au nanoparticles were synthesized according to the previously described procedures [23–25]. The first hydrosol was obtained using the procedure [23] and contained Au nanoparticles with a mean diameter of ~15 nm. The second hydrosol containing Au nanoparticles 5.5 nm in diameter was synthesized according to [24]. Finally, the third hydrosol containing quasi-metallic Au nanoparticles 2–3 nm in diameter was obtained according to the procedure described in [25]. The mean size of the Au nanoparticles in the initial solutions was determined via dynamic light scattering and electron microscopy. The numerical concentration of Au nanoparticles in the first, second, and third hydrosols was 10 ^12^ , 10 ^13^ , and 10 ^15^ particles/cm ^3^ , respectively. 

The Au nanoparticles were negatively charged; their ξ-potentials were as follows: for 2–3 cm particles, –18 ± 7 mV (immediately after synthesis), –25 ± 5 mV (2 days after the synthesis) and –38 ± 5 mV (9 months after the synthesis); for 5 nm particles, –32 ± 4 mV; for 15 nm particles, –44 ± 3 mV. 

The original solutions of Au nanoparticles were stored at 4°C in light-impermeable containers and used 2.5 months following the synthesis. 

A calf thymus depolymerized DNA (Sigma, USA) with a molecular mass of (0.3–0.7) × 10 ^6^ Da after additional purification was used. A synthetic double-stranded polyribonucleotide poly(I)×poly(С) (Sigma, USA; lot 023K4032) was used without additional purification. DNA and poly(I)×poly(С) concentrations in the water–salt solutions were determined spectrophotometrically using the known values of the molar extinction coefficients (ε _max_ = 6,600 М ^–1^ ×cm ^–1^ for DNA and ε _max_ = 4,900 М ^–1^ ×cm ^–1 ^ for poly(I)×poly(С)). 

 Poly(ethylene glycol) samples (PEG; Serva, Germany; molecular mass of 4,000 Da) were used without additional purification. 

The absorption spectra were taken by Cary 100 Scan (Varian, USA) spectrophotometer. The circular dichroism (CD) spectra were recorded using an SKD-2 portable dichrometer. The CD spectra were represented as a dependence of the difference between the intensities of absorption of left- and right-handed polarized light (ΔA; ΔA = (A _L_ – A _R_ )) on the wavelength (λ). 

CLCD of DNA in PEG-containing water–salt solutions were prepared according to the previously described procedure [7]. 

A series of control experiments were carried rut to check the possible interaction between Au nanoparticles and biopolymer molecules (nucleic acids and proteins). 

As has already been mentioned in Introduction, a number of questions pertaining to the behavior of negatively charged small-sized Au nanoparticles under the conditions used were to be answered. Are these Au nanoparticles capable of: 

a) forming aggregates in solutions of low or high ionic strength; 

b) interacting (form complexes) with a neutral polymer (PEG) used to form DNA CLCD particles; 

c) affecting single-stranded nucleic acid molecules in low- or high-ionic-strength solutions; and 

d) affecting double-stranded DNA molecules under conditions that prevent dispersion formation in a PEG-containing water–salt solution. 

**Absorption spectra **

**Fig. 1:**
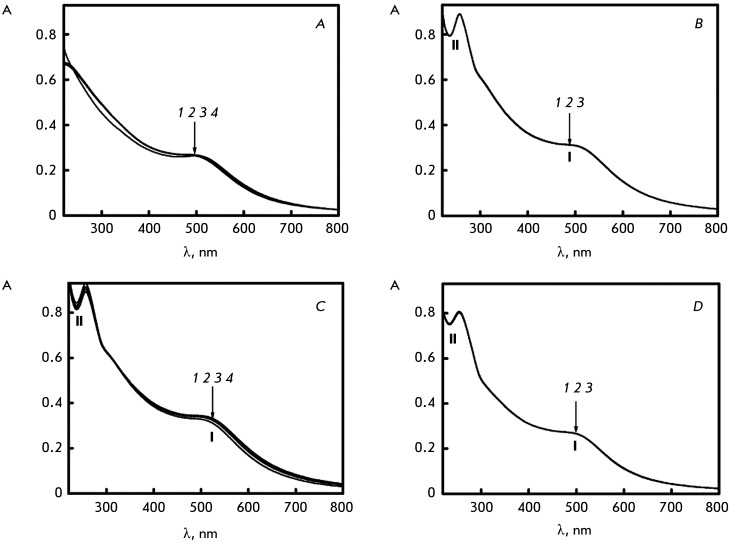
The absorption spectra of Au nanoparticles under various conditions. А. The absorption spectra of Au nanoparticles in a PEG-containing water–salt solution (curves *1–4* ): the PEG-containing solution was treated with Au nanoparticles for: *1* – 1 min; *2* – 6 min; *3* – 17 min; *4* – 280 min; С _PEG_ = 150 mg/ml; 0.27 М NaCl + 1.78×10 ^-3^ М Na ^+^ -phosphate buffer; С _Nano-Au_ = 0.82×10 ^14^ particles/ml. B. The absorption spectra of Au nanoparticles in a water–salt solution of a single-stranded polynucleotide (polyA) (curves *1–3* ): poly(A)-water–salt solution treated with Au nanoparticles for: *1* – 1 min; *2* – 17 min; *3* – 30 min; С _Poly(A)_ = 9 µg/ml; refer to *Fig. 1A* for the other conditions. C. The absorption spectra of Au nanoparticles in a PEG-containing water–salt solution of a single-stranded polynucleotide (polyA) (curves *1–4* ): the poly(A)-PEG-containing solution was treated with Au nanoparticles for: * 1* – 1 min; *2* – 7 min; *3* – 15 min; *4* – 30 min; С _Poly(A)_ = 9 µg/ml; refer to *Fig. 1A* for the other conditions. D. The absorption spectra of Au nanoparticles in a water-salt-DNA-PEG-containing solution of low ionic strength (curves *1–3* ): the DNA-PEG-containing solution was treated with Au nanoparticles for: 1 – 1 min; 2 – 17 min; 3 – 180 min; С _DNA_ = 9 µg/ml; 0.0009 М NaCl; refer to *Fig. 1A* for the other conditions

The absorption spectra of Au nanoparticles recorded at different times after PEG (C _PEG_ = 150 mg/ml) addition to the solution are compared in *Fig. 1A* . It is clear that the absorption spectrum is characterized by a poorly pronounced band (I) at λ ~ 500 nm and a broadband in the short wave spectral region, which is caused by electron transitions both between the d orbitals and the sp hybridized orbitals of Au [26]. The amplitude constancy of the band at λ ~ 500 nm in the absorption spectrum and the absence of either red or blue shifts in this band unambiguously attest to the fact that negatively charged small-sized Au nanoparticles do not tend to aggregate near the surface of PEG molecules under the conditions used. 

*Fig. 1B* shows the absorption spectra recorded at different time intervals after Au nanoparticles addition to the water–salt solution of synthetic single-stranded polynucleotide poly(A). *Fig. 1C* shows the absorption spectra recorded after Au nanoparticles were added to a PEG-containing (C _PEG_ = 150 mg/ml) water–salt solution of the same biopolymer. There are two bands in the absorption spectra in *Figs. 1B,C* : the band in the UV region of spectrum ( **I** ) corresponds to Au nanoparticles; the band in the UV region of spectrum ( **II** ) contains the contribution of the absorption of chromophores in the polynucleotide. The position of these bands and their maxima do not change over time after Au nanoparticles are added to the solutions. 

The absorption spectra of Au nanoparticles recorded at different time intervals after the Au nanoparticles were added to the water–polymer solution (C _PEG_ = 150 mg/ml) of low ionic strength containing double-stranded DNA molecules are shown in *Fig. 1D* . The absorption spectrum contains two bands; the band in the visible region of spectrum ( **I** ) corresponds to Au nanoparticles, whereas that in the UV region of spectrum ( **II** ) corresponds to absorption of DNA chromophores. Phase separation of double-stranded DNA molecules does not happen under the conditions used (ionic strength 0.001 and C _PEG_ = 150 mg/ml); thus, no DNA CLCD are formed. No changes in the amplitudes of both bands are observed under these conditions. 

**Circular dichroism spectra **

The CD spectra of water–salt solutions containing linear double-stranded DNA or poly(I)×poly(C) molecules attest to the fact that treatment of these molecule with Au nanoparticles causes no optical changes in them (spectra are not shown). 

Thus, the absence of any noticeable changes in the amplitude and position of the 500 nm band in the absorption spectra shown in *Fig. 1A* and in the CD spectra indicates that small-sized negatively charged Au nanoparticles neither undergo aggregation in aqueous solutions of low or high ionic strength nor form aggregates near PEG molecules under the selected conditions. Moreover, no changes in the amplitudes of the bands characterizing the optical properties of nitrogen bases or small-sized Au nanoparticles are observed under conditions when there is no phase separation of single-stranded polynucleotide molecules ( *Fig. 1C* ) or double-stranded DNA ( *Fig. 1D* ) and a biopolymer molecule dispersion is not formed [7]. 

The influence of small-sized Au nanoparticles on double-stranded DNA and the poly(I)×poly(C) molecules fixed in the spatial structure of CLCD particles has been investigated with allowance for the results of control experiments. 

## RESULTS AND DISCUSSION 

Before analyzing the effect of Au nanoparticles on double-stranded DNA and the poly(I)×poly(C) molecules fixed in the spatial structure of CLCD particles, let’s provide some illustrations of the structure of the initial liquid-crystalline dispersion particles. In physicochemical terms, each particle in the dispersion is a “droplet” of a concentrated DNA solution, whose structure and properties are determined by the osmotic pressure of the solution [7]. A “droplet” cannot be held in one’s hands or immobilized on a substrate, since the “droplet” structure will change without the osmotic pressure of the solution, and DNA molecules will be converted from their condensed into an isotropic state. Each CLCD particle consists of double-stranded nucleic acid molecules forming its neighboring (so-called quasinematic) layers [7]. *Fiure. 2* illustrates certain features of the quasinematic layer consisting of ordered neighboring double-stranded molecules of nuclear acids (in particular, DNA). In the case of phase separation, the dispersion particles (hence, the quasinematic layer as well) do not contain molecules of a water-soluble polymer (PEG) molecule. There is “free space” both between the neighboring DNA molecules in the same layer and between the DNA molecules in the neighboring layers. The distance between two neighboring DNA molecules in a layer (d) can vary within the 2.5–5.0 nm range, depending on the osmotic pressure of the solution. Under the conditions used (C _PEG_ = 150 and 170 mg/ml), the distance between two DNA molecules determined via an X-ray diffraction analysis of the phases obtained by low-speed precipitation of the initial DNA CLCD particles [7] was 3.6 and 3.2 nm, respectively. DNA molecules ordered in layers retain almost all their diffusion degrees of freedom. Due to the anisotropic properties of DNA molecules, each subsequent quasinematic layer is rotated by a certain angle (approximately 0.1 ^о^ [7]) with respect to the previous one. The rotation gives rise to the helical (cholesteric) structure of a liquid-crystalline dispersion particle. The emergence of this structure can be easily detected according to the abnormal optical activity manifested as a characteristic intense band in the CD spectrum in the region of absorption of DNA chromophores (nitrogen bases). High local concentration of DNA and the ordered arrangement of these macromolecules in a layer provide conditions for a rapid interaction between molecules of various low-molecular-mass compounds (“guests”) with DNA molecules (intercalation between base pairs, fixation in the grooves on the molecule surface, etc.). The distortion of the secondary DNA structure accompanying this interaction affects not only the properties of all quasinematic layers, but also the character of the interaction between them (hence, the structural features of any CLCD particle and its properties as well). Since the properties of the quasinematic layer(s) are determined by the physicochemical properties of DNA CLCD particles, we will use this very term when reporting further results. Finally, complete separation of the chains of double-stranded DNA molecules in a quasinematic layer and their folding into individual random coils is infeasible for steric reasons [27, 28]. 

**Fig. 2:**
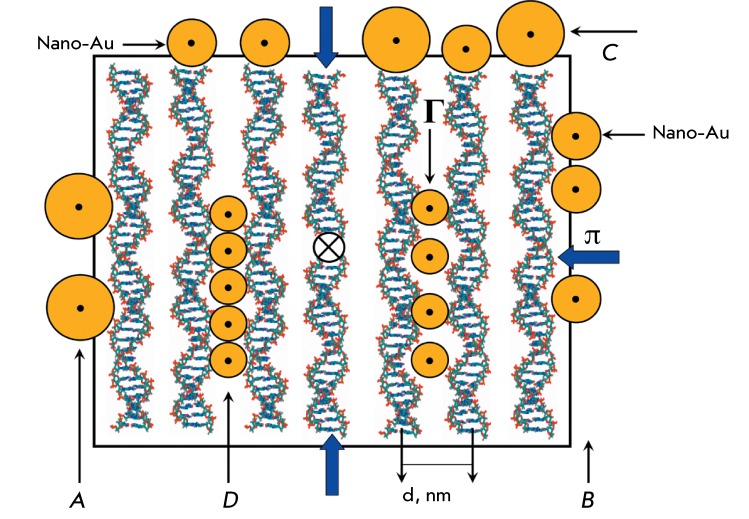
A hypothetical scheme of the arrangement of Au nanoparticles of different sizes ( *A–D* ) near the DNA molecules forming the quasinematic layer. The frame and wide arrows indicate the presence of osmotic pressure (π) in the PEG-containing solution; *d* – distance between the axes of the neighboring DNA molecules

These features of the quasinematic layer allow to hypothesize about the possible mechanisms of fixation of Au nanoparticles (“guests”) near the double-stranded DNA molecules of the quasinematic layer ( *Fig. 2* ). 

First, Au nanoparticles of any size ( *Figs. 2A–C* ) can interact both with the “surface” DNA molecules and with the base pairs of the terminal groups of DNA molecules in the quasinematic layers, thus forming complexes (ensembles) with them [13, 29–31]. 

Second, it is quite possible that Au nanoparticles, whose size is comparable to the distance between the DNA molecules in the quasinematic layer, can diffuse inside the layers ( *Fig. 2D* ), interact with the neighboring DNA molecules within the same quasinematic layer or neighboring quasinematic layers, and form linear clusters. 

One can assume that binding even of a small number of negatively charged Au nanoparticles to DNA molecules (in particular, to the terminal groups in these molecules) results in dipole formation (it should be mentioned there is no need for penetration of Au particles into the quasinematic layer). Dipoles from the neighboring (DNA–Au) complexes within a quasinematic layer, as well as the layers, will tend to be organized in parallel fashion, which can eventually induce a change in the helical twisting of the neighboring quasinematic layers made of DNA molecules. The twist angle between these layers (~ 0.1 ^о^ [7]) can fall to zero, which is equivalent to untwisting of the cholesteric helical structure, and this process will manifest itself as the attenuation of the abnormal band in the CD spectrum of liquid-crystalline dispersion particles. 

It is obvious that although it has no significant effect on the forces (sterical, etc.) that determine the tendency of the neighboring DNA molecules to organize in a parallel fashion, even a small number of negatively charged Au nanoparticles can induce changes in the contributions (in particular, anisotropic contribution to the van der Waals interaction) that control the helical twisting of the neighboring quasinematic layers of DNA molecules. In this case, the helical twisting of the neighboring quasinematic layers will be disturbed and the twist angle between these layers (~ 0.1 ^о ^ [7]) can be equal to zero, which is equivalent to untwisting of the cholesteric helical structure accompanied by attenuation of the abnormal band in the CD spectrum of liquid-crystalline dispersion particles. 

Therefore, it can be expected that if negatively charged Au nanoparticles somehow interact with double-stranded DNA molecules in CLCD particles, this interaction will be accompanied by changes in the abnormal optical activity, which is characteristic for this dispersion. 

It is also quite possible that when neighboring Au particles localize near DNA molecules in a certain fashion, interaction between these nanoparticles can result in the emergence of a surface plasmon resonance band in the absorption spectrum [1, 13, 19]. 

**Changes in circular dichroism spectra caused by the treatment of DNA CLCD particles with Au nanoparticles **

**Fig. 3:**
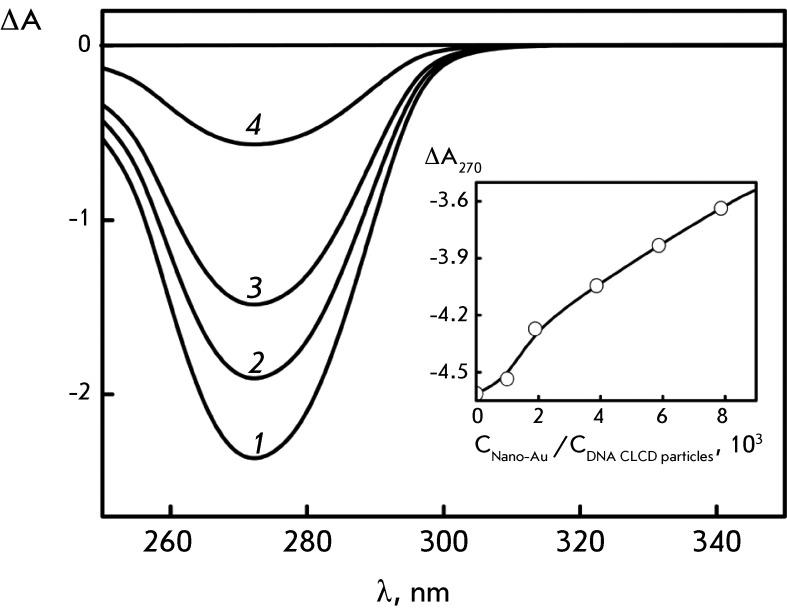
The CD spectra of DNA CLCD treated with Au nanoparticles (2 nm): *1* – С _Nano-Au_ = 0; *2* – С _Nano-Au_ = 0.07×10 ^14^ particles/ml; *3* – С _Nano-Au_ = 0.26×10 ^14^ particles/ml; *4* – С _Nano-Au_ = 0.82×10 ^14^ particles/ml. (Treatment time – 3 h). С _DNA_ = 9 µg/ml; refer to *Fig. 1A* for the other conditions. ∆А = (А _L_ – A _R_ )×10 ^-3^ opt. units; L = 1 cm. Inset: the dependence of the ∆А _270_ value on the С _Nano-Au _ /С _DNA CLCD particles_ ratio obtained for the solution (С _PEG_ = 170 mg/ml) providing maximum abnormal optical activity of DNA CLCD particles is shown as an example

Treatment of DNA CLCD particles with Au nanoparticles results in a decrease in the amplitude of the abnormal negative band in the CD spectrum ( *Fig. 3* ). The fact that the band has a negative sign indicates that right-handed helical double-stranded DNA molecules give rise to a left-handed helical structure of the CLCD particles [7]. 

Due to the effect of Au nanoparticles, the amplitude of the abnormal band in the CD spectrum of DNA CLCD decreases within a rather short period of time. The decrease in the amplitude of the abnormal band in the CD spectrum of DNA CLCD particles becomes pronouncedly stronger as the concentration of Au nanoparticles in the solution increases. It should be mentioned that noticeable changes in the amplitude of the abnormal band in the CD spectrum of DNA CLCD starts at some critical concentration of Au nanoparticles in a solution of approximately 1,000 Au nanoparticles per DNA CLCD particle ( *Fig. 3, inset* ). 

Similar data characterizing the decrease in the abnormal band in the CD spectrum of CLCD formed by synthetic double-stranded poly(I)×poly(C) molecules caused by treatment with Au nanoparticles were presented in [6]. It should be mentioned that the emergence of a positive band in the CD spectrum of this CLCD attests to the fact that the right-handed helices of double-stranded poly(I)×poly(C) molecules form CLCD particles with right-handed twisting of their spatial helical structure. 

The rapid decrease in the amplitude of the band in the CD spectrum of DNA CLCD depends on the size of Au nanoparticles. In particular, if Au nanoparticles are 2 nm in diameter, the amplitude of the abnormal band in the CD spectrum decreases by 75%, whereas when 15-nm diameter nanoparticles are used, it decreases by only 20% [32]. 

The decrease in the amplitude of the band in the CD spectrum of DNA CLCD is also dependent on the temperature of the solution where the dispersion particles are treated with Au nanoparticles [32]. 

In combination with the differences in the efficiency of the changes in the CD spectrum for nanoparticles of different sizes, the scheme shown in *Fig. 2* allows to assume that there are two reasons for the decrease in the abnormal optical activity of DNA CLCD or poly(I)×poly(C) CLCD particles. First, individual Au nanoparticles of any size ( *Figs. 2A–C* ) can interact with the “surface” DNA molecules to yield complexes or linear ensembles (clusters). In this case, small-sized Au nanoparticles can localize in the grooves of the “surface” DNA molecules [31, 33] or form complexes with pairs of DNA nitrogen bases (in particular, with N7 atoms of purines [34–37]). Second, Au nanoparticles whose sizes are comparable to the distance between the DNA molecules in quasinematic layers can diffuse inside the layers to interact with DNA molecules. It is important to mention two aspects here. 1) It was found as early as in the first experiments [13, 38, 39] that Au nanoparticles can form ensembles near the surface of linear single-stranded DNA molecules. Ensemble formation from Au nanoparticles was subsequently shown to be accompanied by the formation of planar suprastructures consisting of repeating double-stranded DNA molecules and Au nanoparticles. These results demonstrate unambiguously that, after they interact with Au nanoparticles, DNA molecules tend to form planar suprastructures [30, 39, 40], despite the fact that the original DNA molecules possess anisotropic properties [7]. 2) In case of CLCD particles of double-stranded DNA molecules, formation of an ensemble even of a small number of Au nanoparticles on “surface” DNA molecules or near DNA molecules in quasinematic layers will result in changes in the character of the interaction between neighboring quasinematic layers. This can result in the attenuation of the helical twisting of the neighboring layers; i.e., the spatial helical structure of CLCD particles will untwist. 

With allowance for the formation of planar structures considered above, it can be stated that Au nanoparticles (in case of CLCD particles) initiate a parallel (rather than helical) arrangement of the neighboring quasinematic layers of DNA molecules. 

Regardless of the aforementioned reasons, combination of the control experiments ( *Fig. 1* ) and the results obtained ( *Fig. 3* ) allows one to suggest that the action of Au nanoparticles is directed towards the double-stranded DNA molecules fixed in the CLCD particles. Meanwhile, the rapid decrease in the abnormal band in the CD spectrum can be attributed to binding of an appreciably small number of Au nanoparticles to the DNA molecules in CLCD particles. This process is accompanied by the disturbance of the helical mode of ordering in the neighboring quasinematic layers; i.e., Au nanoparticles induce a transition similar to the known cholesteric → nematic transition [7]. 

Thus, the changes in the CD spectra of DNA CLCD (or poly(I)×poly(C) CLCD) indicate that Au nanoparticles of different sizes can interact with the double-stranded molecules of nucleic acids or synthetic polynucleotides within CLCD particles (the efficiency of the interaction may vary), although most of the details of the mechanism underlying the interaction remain unclear. 

**Changes in the absorption spectra caused by the treatment of DNA CLCD particles with Au nanoparticles **

**Fig. 4:**
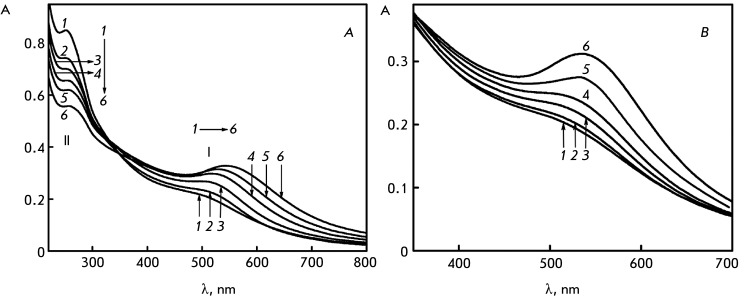
The absorption spectra of DNA CLCD (А) and poly(I)×poly(C) CLCD (B) treated with Au nanoparticles during various time intervals. А. Treatment for: 1 – 0 min; 2 – 1 min; 3 – 8 min; 4 – 21 min; 5 – 36 min; 6 – 100 min. B. Treatment for: 1 – 2 min; 2 – 30 min; 3 – 50 min; 4 – 85 min; 5 – 115 min; 6 – 256 min. С _DNA_ = 9 µg/ml; С _Poly(I)×poly(C)_ = 9 µg/ml; refer to *Fig. 1A* for the other conditions

The analysis of the absorption spectra of Au nanoparticles permits an assessment of the size of the ensembles formed by these particles under various conditions [41–44]. 

Noticeable changes both in the visible and in the UV spectral regions are observed after DNA CLCD particles are treated with small-sized Au nanoparticles ( *Fig. 4A* ). This treatment is primarily accompanied by changes in band ( **I** ) at 550 nm (SPR band) [41, 42]. *Fig. 4B* shows the data obtained by treating CLCD formed by poly(I)×poly(C) molecules (their particles are characterized by left-handed twisting of the spatial structure) with Au nanoparticles. It is clear that treatment with Au nanoparticles in this case is also accompanied by the development of the plasmon effect. 

The emergence of the SPR band is responsible for the pink-violet color of the solution containing DNA CLCD or poly(I)×poly(C) CLCD and treated with Au nanoparticles. The control experiments ( *Fig. 1* ) have demonstrated that the band at ~505 nm is poorly pronounced in the absorption spectrum of Au nanoparticles and remains almost unchanged when solvent properties are varied. The intensity of the SPR band gradually increased over time; its maximum shifted from λ ~ 505 to ~ 550 nm. Meanwhile, the amplitude of band ( **II** ) in the UV region of the spectrum corresponding to the absorption of DNA chromophores decreases over time. It should be also mentioned that according to theoretical calculations [45], similar changes in bands ( **I** ) and ( **II** ) in the absorption spectrum are responsible for the increase in the volume fraction of Au nanoparticles in the ensemble formed by these particles. 

**Fig. 5:**
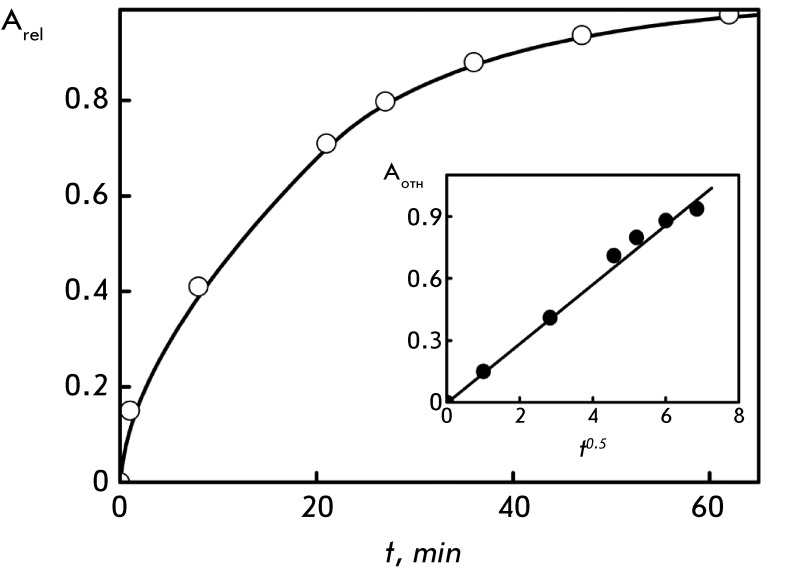
Time dependence of the amplitude of the SPR band (λ = 550 nm) for Au nanoparticles interacting with DNA CLCD. С _DNA_ = 9 µg/ml; refer to *Fig. 1A * for the other conditions. Inset: dependence of the amplitude of the SPR band (λ = 550 nm) for Au nanoparticles interacting with DNA CLCD on t ^0.5^ value (t is given in min)

**Fig. 6:**
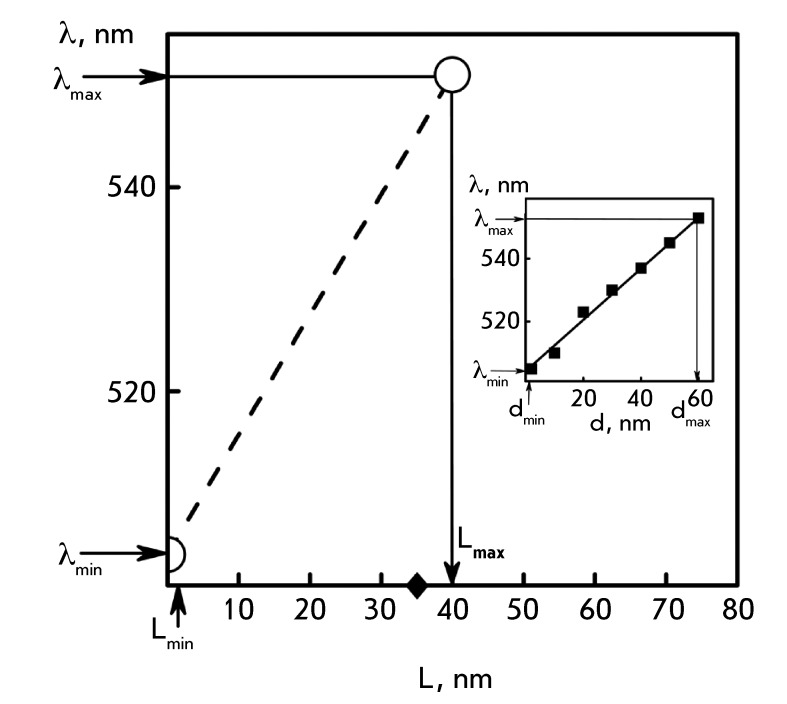
Position of the surface plasmon resonance (SPR) peak as a function of the size of the linear clusters of Au nanoparticles, which are formed in the spatial structure of DNA CLCD particles. Symbol (♦) shows the data for the linear cluster of Au nanoparticles formed within the spatial structure of poly(I)×poly(C) CLCD particles. Inset: dependence of the position of the SPR peak on the diameter of spherical Au nanoparticles (the average data are taken from [40–43])

It is characteristic that the treatment of DNA CLCD particles with Au nanoparticles 5 and 15 nm in diameter does not result in any changes in the absorption spectra of these nanoparticles. This fact gives ground to hypothesize that there are noticeable differences in the mechanisms of action of small- and large-size Au nanoparticles on DNA CLCD particles. Indeed, it can be seen from the scheme shown in *Fig. 2* that Au nanoparticles of any size ( *A–C* ) can localize near the “surface” DNA molecules of the quasinematic layer and form linear ensembles. Formation of these ensembles even from a small number of Au nanoparticles can be accompanied by the enhancement of the SPR band [1]. 

It is important to note that the emergence of the plasmon effect does not require direct contact between neighboring Au nanoparticles, and the plasmon effect can be observed as long as the distance between the neighboring nanoparticles is shorter than the wavelength of the incident light [1]. 

The absence of changes in the absorption spectrum of CLCD particles after they are treated with Au nanoparticles 5 and 15 nm in diameter, in combination with the scheme given in *Fig. 2* , allows one to assume that in addition to the known fact that Au nanoparticles are ordered near single-stranded or linear double-stranded DNA molecules [29–31, 39, 40], there is a different mechanism of arrangement of small-sized Au nanoparticles in DNA CLCD particles. 

The evolution of the SPR band during the treatment of DNA CLCD with Au nanoparticles lasts for ~100 min ( *Fig. 5* ); then, its saturation occurs. The direct proportional dependence between the amplitude of the SPR band (until the saturation point) and the *t* ^0.5^ value is retained. Under the assumption that the amplitude of the SPR band is associated with the concentration of Au nanoparticles in the resulting ensemble, the dependence shown in the inset ( *Fig. 5* ) represents the diffusion of Au nanoparticles [46] into the quasinematic layers of CLCD particles. 

*Fig. 6* (inset) shows the dependence between the position of the SSR band maximum on the size of spherical Au nanoparticles, which was constructed by averaging the published data [40–43]. It was demonstrated by comparing the results shown in *Fig. 4* with this dependence that the size of Au nanoparticles after their binding to DNA CLCD particles has the potential to increase from 2 to ~60 nm. Although this estimation is not consistent enough, since the dependence characterizes the properties of Au nanoparticles of spherical shape, it still can be used for comparative assessment of the size of Au nanoparticles formed under various conditions. 

**Fig. 7:**
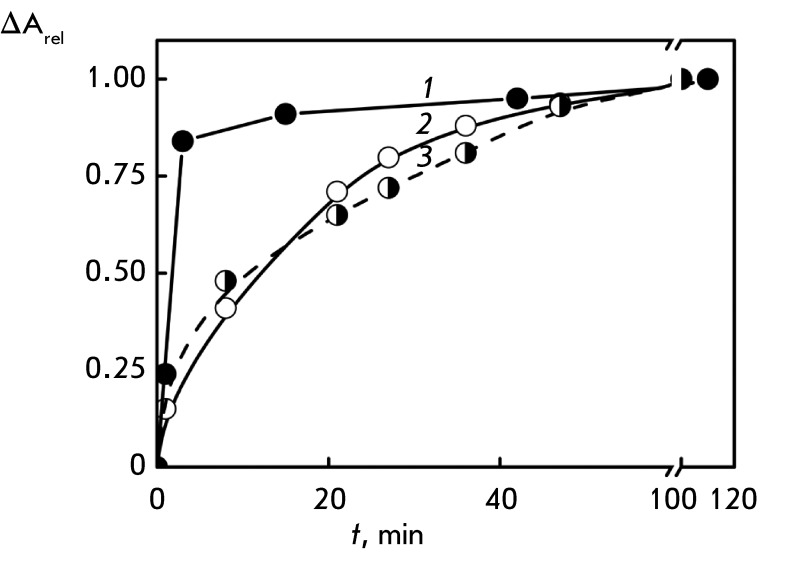
Dependence of the CD band amplitude (λ = 270 nm, curve *1* ), location of the SPR peak (λ = 550 nm, curve *2* ), and band at λ = 270 nm (curve *3* ) in the absorption spectrum of DNA CLCD on the time of treatment with Au nanoparticles. С _DNA_ = 9 µg/ml; refer to *Fig. 1A* for other conditions

**Fig. 8:**
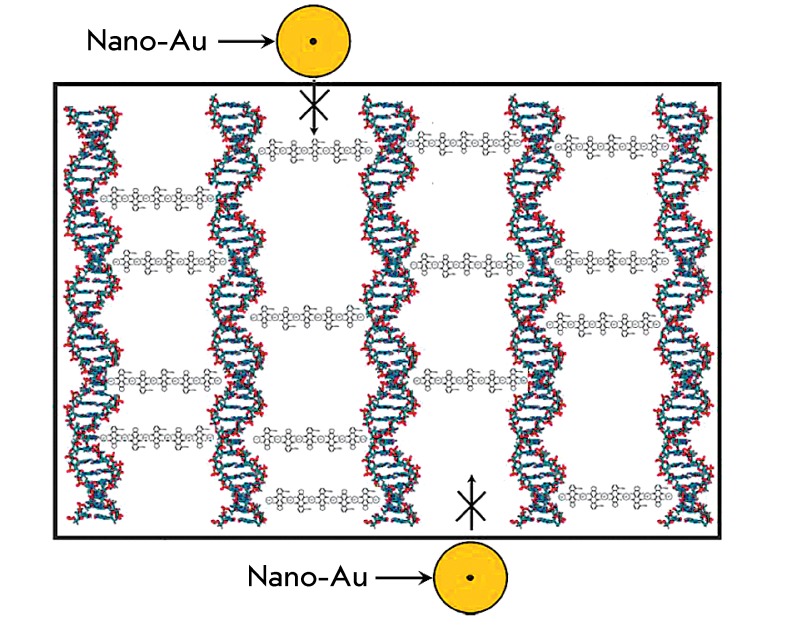
A hypothetical structure of the quasinematic layer in a DNA nanoconstruction. The neighboring DNA molecules forming the quasinematic layer are “cross-linked” via nanobridges, which do not allow Au nanoparticles to penetrate into the layer and to form clusters in the «free space» between DNA molecules (the probability of their interaction with the “surface” DNA molecules remains unchanged). The frame means the presence of osmotic pressure in the PEG-containing solution

The results presented in [6] and characterizing low-angle X-ray scattering from the phases formed by DNA CLCD particles treated with Au nanoparticles allow one to make a more accurate estimation of the particle size. These results indicate that linear clusters of Au nanoparticles with a maximum size of 40 nm are formed within the structure. The SPR band is characterized by a maximum at λ ~ 550 nm [6]. The dependence of the position of the SPR peak on the linear size of Au clusters ( *Fig. 6* ) can be constructed using these findings (i.e., it directly describes Au nanoparticle clusters formed upon interaction between Au nanoparticles and particles of CLCD of various nucleic acids). It is clear that the actual size of the resulting ensemble (the linear cluster of Au nanoparticles) for DNA increases from 2 to 40 nm. Treatment of poly(I)×poly(C) CLCD with Au nanoparticles results in an increase in the size of Au nanoparticles up to 34 nm (these data are indicated by ♦ symbol on the X axis in *Fig. 6* ). 

It should also be mentioned that the size of the linear clusters of Au nanoparticles was never higher than 40 nm under the experimental conditions used (negatively charged Au particles, high ionic strength of solutions [47, 48], etc.). 

The results presented in *Figs. 5 * and * 6* enable one to analyze more thoroughly the diffusion mechanism of formation of Au nanoparticle clusters. Since the concentration of Au nanoparticles “outside” DNA CLCD particles is higher than that “inside” (i.e., between the quasinematic layers), the concentration gradient induces the emergence of a diffusion flow of Au nanoparticles. The flow stops when the concentrations “outside” and “inside” DNA CLCD particles become equal. If the characteristic time of attaining this equilibrium is *t* , the size of a cluster formed by the diffused Au nanoparticles increases as the square root of time (i.e., as *t* ^0.5^ ). One can expect this process to be hindered by the lower translational entropy value of the Au nanoparticles concentrated inside a cluster (i.e., in the «free space» between the quasinematic layers) as compared to that of the Au nanoparticles which are freely distributed over the solution. Since the entropy factor is proportional to *k*_B_ *T* , the size of the Au nanoparticle clusters formed in nucleic acid CLCD particles will decrease with increasing temperature of the solution. 

Thus, in our case the shift in the position of the SPR band is associated with the size of the linear Au nanoparticle clusters within nucleic acid CLCD particles formed under various conditions rather than with an increase in the true size of individual Au nanoparticles. The problem of estimating Au nanoparticles in a cluster based on the results of optical changes remains unsolved, since the position of the SPR peak depends on the number and distance between the Au nanoparticles in a cluster, the dielectric permittivity of the medium, and other parameters [19]. 

**Fig. 9:**
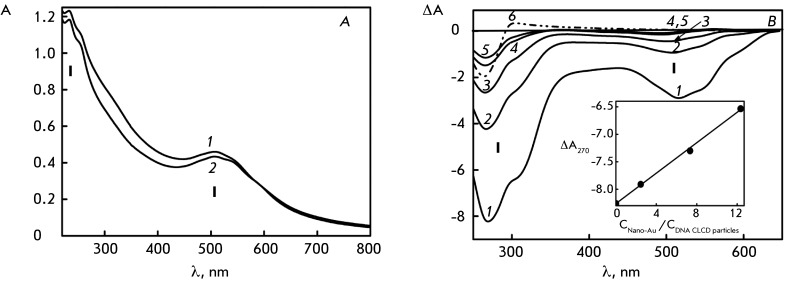
The absorption (А) and CD (B) spectra of a DNA nanoconstruction treated with Au nanoparticles. A – Treatment for: 1 – 0 min; 2 – 100 min. B – Treatment for: 1 – 0 min; 2 – 10 min; 3 – 25 min; 4 – 55 min; 5 – 100 min. С _DNA_ = 5 µg/ml; С _PEG_ = 150 mg/ml; С _DAU_ = 3.2×10 ^-5^ М; С _Cu_ = 1×10 ^-5^ М; refer to *Fig. 1A* for the other conditions. Inset: ∆А _270_ value as a function of the С _Nano-Au _ /С _DNA CLCD particles _ ratio in the solution

With allowance for these results and the hypothetic scheme ( *Fig. 2* ) showing all possible ways for Au nanoparticles to bind to the DNA molecules fixed in the structure of CLCD particles, as well as for the changes in the amplitudes of the bands localizing in different regions of the absorption spectrum ( *Fig. 4* ), which was not observed in the control experiments with single-stranded polynucleotide or double-stranded DNA molecules under conditions impeding their condensation ( *Fig. 1* ), one can consider that small-sized Au nanoparticles (2 nm) can form linear clusters in CLCD particles. 

Although capable of interacting with the “surface” DNA molecules ( *Figs. 2A,B* ) or terminal groups of DNA molecules ( *Fig. 2C* ) in quasinematic layers, Au nanoparticles 5 and 15 nm in diameter are too large to be incorporated between the DNA molecules in these layers. 

*Fig. 7* shows the curves that characterize the rate of changes in the amplitude of the abnormal band in the CD spectrum of DNA CLCD, of the SPR band, and of the band located in the UV region of the absorption spectra after the dispersion is treated with small-sized Au nanoparticles. It is clear that the treatment of DNA CLCD with Au nanoparticles is accompanied by two simultaneous processes: a fast decrease in the abnormal optical activity of DNA CLCD and a slower evolution of the SPR band. The process recorded on the basis of the changes in the abnormal band in the CD spectrum lasts 10–15 min, whereas the evolution of the SPR band requires approximately 60 min. 

Thus, in addition to the fast interaction between Au nanoparticles (of any size) and DNA CLCD particles (which is required to change their abnormal optical activity to a certain extent), incorporation of small-sized Au nanoparticles in the structure of CLCD particles yielding Au nanoparticle clusters is also possible. 

**Absorption and CD spectra obtained for CLCD particles with DNA molecules cross-linked by nanobridges treated with Au nanoparticles **

An important issue is where the Au nanoparticle clusters localize. It can be assumed that small-sized Au nanoparticles diffuse into the “free space” between neighboring DNA molecules in the quasinematic layers of CLCD particles to cluster there. This process is accompanied by the emergence and evolution of the SPR band ( *Fig. 4* ). 

In order to verify this assumption, the “free space” between the neighboring DNA molecules in CLCD particles was filled with appreciably strong nanobridges [49] consisting of alternating antibiotic molecules and copper ions ( *Fig. 8* ). This process resulted in the formation of a DNA nanoconstruction. In this case, the “free space” becomes inaccessible for diffusion and clustering of Au nanoparticles. 

If the assumption about the localization of Au nanoparticle clusters is valid, treatment of the DNA nanoconstruction with Au nanoparticles will not result in any changes in the bands located both in the UV and visible regions of the absorption spectrum. Indeed, it is clearly seen in *Fig. 9A * that no significant changes in the absorption spectrum of the nanoconstruction obtained from CLCD particles due to the formation of nanobridges between DNA molecules are observed and that SPR band ( **I** ) does not evolve in this case. Meanwhile, band ( **II** ) in the UV region of the spectrum remains virtually intact. This means that small-sized Au nanoparticles cannot insert themselves between the neighboring DNA molecules in quasinematic layers, since the “free space” is occupied by nanobridges [49]. 

**Fig. 10:**
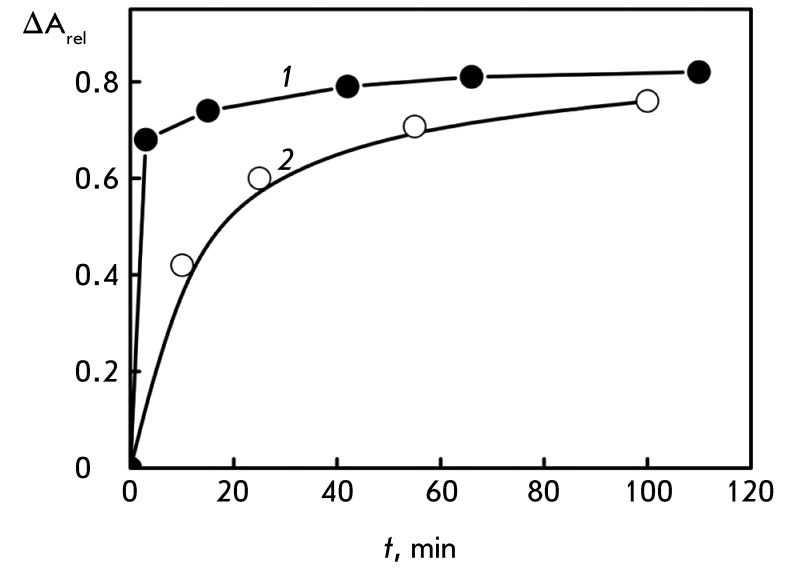
Kinetic curves characterizing the change in the abnormal optical activity of the initial DNA CLCD (curve *1* ) and DNA nanoconstruction (curve *2* ) induced by treatment with Au nanoparticles (2 nm). Curve 1- С _DNA_ = 9 µg/ml; С _DNA_ = 5 µg/ml; С _PEG_ = 150 mg/ml; С _DAU_ = 3.2×10 ^-5^ М; С _Cu_ = 1×10 ^-5^ М; refer to *Fig. 1A * for the other conditions

One can focus on the fact that the nanobridges increase the rigidity of the spatial structure of the nanoconstructions [49]. Hence, although “surface” DNA molecules in particles of nanoconstructions are available for interacting with Au nanoparticles, the untwisting process (in the case when a nanoconstruction is treated with Au nanoparticles) accompanied by a decrease in the abnormal band in the CD spectrum of the nanoconstructions will require a longer period of time and can be terminated even at a smaller “depth” of this process. The CD spectra of the original DNA CLCD (dashed curve *6* ), DNA nanoconstruction (i.e., CLCD with the neighboring DNA molecules cross-linked via nanobridges; curve *1* ), and the same nanoconstruct treated with Au nanoparticles (curves *2–5* ) are compared in *Fig. 9B* . It is clear that the formation of a DNA nanoconstruction from the original CLCD is accompanied by amplification of the band in the UV region and the emergence of an additional band in the visible region of the spectrum, which is caused by the formation of nanobridges containing chromophores absorbing within this wavelength range [49]. The amplification indicates that the twist angle of the neighboring quasinematic layers increases due to the formation of nanobridges [7]. After the nanoconstruct is treated with Au nanoparticles at a high concentration (С _Nano-Au_ = 0.82 × 10 ^14^ particles/ml), the amplitude of the bands in the UV and visible regions of the spectrum decreases despite the fact that the absorption spectrum does not contain the SPR band. 

*Fig. 10* shows a comparison of the kinetic curves characterizing the changes in the abnormal optical activity caused by treatment of the original DNA CLCD and DNA nanoconstructions with Au nanoparticles. It is clear that the depth and rates of these processes are different for the original DNA CLCD and DNA nanoconstructions, which supports the thesis that the bridges play a stabilizing role. 

The results shown in *Fig. 9* additionally demonstrate that small-sized Au nanoparticles can interact with the “surface” molecules of double-stranded DNA, thus inducing the cholesteric → nematic transition, even if nanobridges form between the neighboring DNA molecules, but cannot diffuse between DNA molecules in the quasinematic layers, since the “free space” is filled with nanobridges. 

Thus, the SPR band can emerge and evolve only if there is “free space” between DNA molecules in quasinematic layers. It is in this very space that Au nanoparticle clusters are formed. 

We previously demonstrated that the interaction between Au nanoparticles and the “surface” DNA molecules in CLCD particles induces changes in the helical spatial distribution of neighboring quasinematic DNA layers (i.e., formation of the nematic structure). It is possible that the probability of one (or several) right-handed helical double-stranded DNA molecule rotating 180 ^о^ with respect to its neighbor(s) due to rotational diffusion in the quasinematic layers located at nanodistances increases at this very moment. In this case, the reactive groups of a DNA molecule (1) localize in the “free space” facing the identical groups of its neighbor (2), which can be referred to as a type of face-to-face phasing of the reactive groups of DNA molecules. Therefore, clustering of negatively charged Au nanoparticles in the “free space” between DNA molecules ( *Fig. 2* ) may result from two processes. First, Au nanoparticles may diffuse into the “free space” between the neighboring “phased” DNA molecules (1 and 2) (in this case, it is a one-dimensional diffusion of Au nanoparticles between these DNA molecules). Second, the interaction between a DNA particle in the quasinematic layer and a negatively charged small-sized Au nanoparticle can be conditionally regarded as the equivalent interaction between a plane and a spherical particle [50]. In this case, the interaction of the Au nanoparticle can be determined by the so-called Casimir effect [51–54]. 

For either version of the processes discussed above (provided that the experimental conditions are fixed), one can assume that Au nanoparticles can form linear clusters between DNA molecules (direct contact between neighboring Au nanoparticles in clusters can be absent) [55]. The clustering of Au nanoparticles is accompanied by the evolution of the SPR band. 

Thus, different processes can determine “sliding” (“retraction”) of Au nanoparticles into the “free space” between neighboring DNA molecules in quasinematic layers. 

Thus, if one accepts the hypothesis of the ordering mechanism of negatively charged Au nanoparticles in quasinematic layers, it becomes clear why small-sized Au nanoparticles form clusters only in CLCD particles comprising double-stranded molecules of nucleic acids or synthetic polyribonucleotides (poly(I)×poly(C))., 

## CONCLUSIONS 

These findings demonstrate that small-sized Au nanoparticles form clusters in the “free space” between the neighboring double-stranded DNA molecules fixed in the spatial structure of CLCD particles. This conclusion allows one to regard a DNA CLCD particle as a matrix that specifically adsorbs small-sized Au nanoparticles and provides conditions for the formation of linear clusters from these nanoparticles. The cytotoxicity of Au nanoparticles can presumably be attributed to their tendency to cluster. 

## Abbreviations

DAU: daunomycin
CD: circular dichroism
Au nanoparticles (nano-Au): gold nanoparticles
SPR: surface plasmon resonance
PEG: poly(ethylene glycol)
UV region: ultraviolet region
CLCD: cholesteric liquid-crystalline dispersion
